# Preliminary exploration of differentially expressed circular RNAs for discriminating atrial fibrillation-related stroke

**DOI:** 10.3389/fcvm.2026.1886593

**Published:** 2026-09-23

**Authors:** Zhong-bao Ruan, Fei Wang, Shu-yi Cong, Xiao-lin Sun

**Affiliations:** 1 Department of Cardiology, the Affiliated Taizhou People’s Hospital of Nanjing Medical University, Taizhou School of Clinical Medicine, Nanjing Medical University, Taizhou, China; 2 Department of Cardiology, Rizhao International Heart Hospital, Rizhao, China

**Keywords:** atrial fibrillation, biomarkers, circular RNAs, discriminate, stroke

## Abstract

**Background:**

Few studies have investigated the detection of circular RNAs (circRNAs) in peripheral blood to discriminate stroke risk in atrial fibrillation (AF). The aim of this study is to identify differentially expressed circRNAs in AF-related stroke in peripheral blood and provide a possible new biomarker for discriminating AF-related stroke.

**Methods:**

A total of 23 patients with AF-related stroke and 23 AF patients hospitalized at Taizhou People's Hospital between August 2020 and December 2020 were enrolled in the study. Peripheral blood samples from three AF-related stroke patients and three AF patients were randomly selected for bioinformatics analysis based on whole-transcriptome sequencing technology. Quantitative real-time polymerase chain reaction (qRT-PCR) was used to validate the microarray hybridization results in the remaining 20 AF-related stroke patients and 20 AF patients. CircRNA–miRNA networks were constructed using Cytoscape 3.4.0 (http://cytoscape.org/). The discriminative effect of differentially expressed circRNAs on AF-related stroke was explored using ROC curves.

**Results:**

A total of 15 circRNAs were identified as differentially expressed between AF-related stroke patients and AF patients, comprising 8 upregulated and 7 downregulated circRNAs. Differentially expressed circRNA-related signaling pathways were mainly involved in signal transduction, infectious diseases, and immune system processes. circRNA_08148, circRNA_12852, and circRNA_00199 emerged as the top three circRNAs with the highest number of binding nodes in the circRNA–miRNA networks. qRT-PCR validation confirmed significant differential expression for three upregulated circRNAs (*p* < 0.05 or *p* < 0.01 for circRNA_00199, circRNA_06049, and circRNA_12852) and two of the three downregulated circRNAs (*p* < 0.05 or *p* < 0.01 for circRNA_11414 and circRNA_09151). circRNA_12852 was identified as an independent risk factor for stroke in AF patients after adjusting for the D-dimer level and neutrophil-to-lymphocyte ratio (odds ratio (OR): 1.80, 95% CI: 1.150–2.817, *p*-value: 0.01). High expression of circRNA_12852 demonstrated some discriminative value for AF-related stroke (area under the receiver operating characteristic curve (AUC): 0.810, 95% CI: 0.662–0.958, *p*-value: 0.0008).

**Conclusions:**

Compared with AF patients, those with AF- related stroke exhibited differentially expressed circRNAs. circRNA_08148, circRNA_12852, and circRNA_00199 emerged as the top three circRNAs with the highest number of binding nodes within the co-expression network. circRNA_12852 was identified as an independent risk factor for discriminating AF-related stroke.

## Introduction

Atrial fibrillation (AF) is one of the most common sustained arrhythmias, with an estimated prevalence of 1%–4% in adults (1). Adults face a one-in-five lifetime risk of developing atrial fibrillation (2). Stroke is the second leading cause of both disability and death worldwide, imposing a serious burden on individuals and society (3). Emerging evidence indicates that AF is a major cause of ischemic stroke in patients with a CHA_2_DS_2_-VASc score ≥2 (males) or ≥3 (females), accounting for up to 23.7% of all strokes (4). As reported in the Oxford Vascular Study (OXVASC), nearly 43.9% of ischemic strokes were related to AF (5). Therefore, the evaluation of thromboembolism is an important component of the treatment strategy for patients with AF. The CHA_2_DS_2_-VASc score is recommended as an evaluation tool for stroke risk in patients with non-valvular AF. However, this score does not include some potential stroke risk factors, such as the blood inflammation index, N-terminal pro-B-type natriuretic peptide (NT-proBNP), and renal function, and offers only moderate predictive ability for stroke (6). Actively identifying markers that can predict AF-related stroke at an early stage will facilitate timely intervention, improve treatment strategies, and enhance the prognosis.

Circular RNAs (circRNAs) are non-coding RNAs with regulatory functions. Their molecular structure consists of closed loops and is not affected by RNA exonucleases, which makes their gene expression more stable (7). Abnormal expression of circRNAs may cause disease. Notably, few studies exist on the relationship between circRNAs and AF-related stroke. Previous studies have found that circRNAs are differentially expressed in the peripheral blood of patients with AF compared with healthy adults, suggesting that the circRNAs-hsa-miR-328 network may play an important role in the pathophysiology and mechanism of AF (8). However, to the best of our knowledge, research on circRNA detection in peripheral blood to discriminate stroke risk in AF remains scarce. There is no doubt that further exploration of circRNAs can deepen our understanding of the pathophysiology and mechanism of AF and enable the screening of AF-related stroke biomarkers.

In the present study, we aimed to identify differentially expressed circRNAs in AF-related stroke using microarray analysis and explore the theoretical basis of the mechanism underlying AF-related stroke. Furthermore, we provided a bioinformatic analysis of the differentially expressed circRNAs and identified circRNAs associated with AF-related stroke. This study may provide novel biological predictive markers and treatment targets for AF-related stroke.

## Materials and methods

### Study population and specimen collection

A total of 23 AF-related stroke patients and 23 AF patients hospitalized at the Affiliated Taizhou People's Hospital of Nanjing Medical University between August 2020 and December 2020 were enrolled in the study. The diagnosis of AF was established based on the criteria outlined in the 2020 ESC Guidelines for the management of atrial fibrillation, developed in collaboration with the European Association for Cardio-Thoracic Surgery (EACTS) (9). AF-related stroke was defined by the following criteria: (1) acute ischemic stroke confirmed by relevant brain imaging within 7 days of symptom onset; (2) AF previously or newly diagnosed via electrocardiography or 24-h Holter monitoring after the index stroke; and (3) fulfillment of the Trial of Org 10172 in Acute Stroke Treatment (TOAST) diagnostic criteria for cardiogenic cerebral infarction (10). This study was approved by the Ethics Committee of the Affiliated Taizhou People's Hospital of Nanjing Medical University, and written informed consent was obtained from all subjects before entering this experiment.

### Identification of differentially expressed circRNAs

Approximately 10 mL of blood was drawn from each patient into ethylenediaminetetraacetic acid (EDTA) anticoagulant tubes. Total RNA was extracted from peripheral blood monocytes using the TRIzol method (Invitrogen, Carlsbad, CA, USA). Microarray hybridization (Outdo Biotech, Shanghai, China) was used to screen for differentially expressed circRNAs in three AF-related stroke patients and three AF patients as previously described (11), and the miRanda database was used to predict their target genes. Gene Ontology (GO) and Kyoto Encyclopedia of Genes and Genomes (KEGG) analyses were performed to identify the functions of differentially expressed genes and related pathways. Quantitative real-time polymerase chain reaction (qRT-PCR) was used to validate microarray hybridization results in 20 AF-related stroke patients and 20 AF patients. For qRT-PCR, 1 µL of cDNA was added to 10 µL of SYBR-Green Gene Expression Master Mix (Applied Biosystems, Inc., Foster City, CA, USA), 8 µL of diethyl pyrocarbonate (DEPC)-treated water, and 0.5 µL of reverse and forward primers. The expression of target circRNAs was determined using GAPDH (Sangon Biotech, Shanghai, China) as a housekeeping gene and calculated by the 2^−ΔΔCt^ method. circRNA and miRNA networks were further constructed using Cytoscape 3.4.0 (http://cytoscape.org/).

### Statistical analysis

SPSS 23.0 and GraphPad Prism 9.0 software were used for statistical analyses. Missing data were processed prior to analysis. For continuous variables with a missing rate below 5%, mean imputation was used to retain the statistical characteristics of the data; for variables with a missing rate ranging from 5% to 20%, regression imputation was used. For continuous variables, the Shapiro–Wilk test was used to assess normality. Data following a normal distribution were expressed as mean ± standard deviation (SD). The qualitative data were expressed as 1/2 quantiles (1/4 quantile to 3/4 quantile), and the categorical data were expressed as numbers and percentages. The independent-samples *t*-test, chi-square test, or Fisher’s exact test was used, respectively. Binary logistic regression analysis was used to explore the risk factors for AF-related stroke. A receiver operating characteristic (ROC) curve was further used to assess the predictive efficacy of circRNAs for AF-related stroke.

## Results

### Comparison of clinical data between AF-related stroke patients and AF patients

A total of 46 AF patients were enrolled, of whom 6 were used for microarray hybridization, and 40 were used for qRT-PCR. As presented in Table 1, there was a statistically significant difference in D-dimer levels and the neutrophil-to-lymphocyte ratio (NLR) (the ratio of absolute peripheral neutrophil count to absolute lymphocyte count) between AF-related stroke patients and AF patients.

**Table 1:** Baseline characteristics of the study population.

Clinical parameters	Total (*n* = 46)	AF-related stroke (*n* = 23)	AF (*n* = 23)	*p*-value
Age, years	74.5 (67.3–80.0)	71.0 (61.0–71.0)	75.5 (68.5–80.8)	0.157
Male patients (*n*, %)	23 (50.0)	13 (56.5)	10 (43.5)	0.523
Hypertension, *n* (%)	24 (52.2)	12 (52.2)	12 (52.2)	1.000
Diabetes, *n* (%)	9 (19.6)	6 (26.1)	3 (13.0)	0.451
CHD, *n* (%)	5 (10.9)	2 (8.7)	3 (13.0)	1.000
Heart failure, *n* (%)	7 (15.2)	2 (8.7)	5 (21.7)	0.407
CHA_2_DS_2_-VASc score	2.2 ± 1.5	3.1 ± 1.3	1.3 ± 1.2	<0.001
BMI (kg/m^2^)	23.5 ± 3.6	23.4 ± 3.4	23.5 ± 3.9	0.976
LVEF (%)	59.0 (53.0–66.0)	58.5 (54.0–65.5)	59.0 (46.8–66.0)	0.925
LA (mm)	47.5 ± 7.7	46.1 ± 8.4	48.9 ± 6.7	0.245
TC (mmol/L)	3.6 ± 1.1	3.9 ± 1.1	3.3 ± 1.0	0.098
TG（mmol/L）	1.1 (0.8–1.4)	1.1 (0.7–1.6)	1.0 (0.8–1.4)	0.602
LDL (mmol/L)	2.2 ± 0.8	2.3 ± 0.9	2.0 ± 0.7	0.225
Cr (µmol/L)	80.1 (63.2–97.6)	79.0 (61.9–89.5)	82.4 (67.8–100.6)	0.265
UA (µmol/L)	397.4 ± 140.6	359.7 ± 149.7	435.2 ± 123.2	0.090
D-dimer (µg/L)	905.0 (332.5–2,102.5)	1,385.0 (782.5–2,480.0)	435.0 (262.5–1,637.5)	0.015
NLR	3.5 (2.6–5.5)	4.4 (3.2–6.5)	2.9 (2.4–5.0)	0.026
Anticoagulant therapy, *n* (%)	39	19	20	1.271
Antiplatelet therapy, *n* (%)	7	4	3	1.271

CHD, coronary heart disease; BMI, body mass index; LVEF, left ventricular ejection fraction; LA, left atrium; TC, total cholesterol; TG, triglyceride; LDL, low-density lipoprotein; Cr, creatinine; UA, uric acid; NLR, neutrophil-to-lymphocyte ratio.

### Analysis of differentially expressed circRNAs by microarray

A total of 15 circRNAs were identified as differentially expressed between AF-related stroke patients and AF patients (fold change >2, and *p* < 0.05), comprising 8 upregulated and 7 downregulated circRNAs (Table 2 and Figure 1).

**Table 2:** Differentially expressed circRNAs between AF-related stroke patients and AF patients.

circRNA_id	FC	log_2_FC	*p*-value	Differential expression
circRNA_00199	inf	inf	0.026	Up
circRNA_03650	2.583	1.367	0.037	Up
circRNA_06049	inf	inf	0.049	Up
circRNA_11361	2.902	1.537	0.049	Up
circRNA_11730	inf	inf	0.001	Up
circRNA_11751	inf	inf	0.041	Up
circRNA_12559	inf	inf	0.009	Up
circRNA_12852	10.929	3.450	0.044	Up
circRNA_02806	0	−Inf	0.040	Down
circRNA_07060	0	−Inf	0.043	Down
circRNA_08148	0.100	−3.317	0.047	Down
circRNA_09151	0	−Inf	0.026	Down
circRNA_11414	0	−Inf	0.036	Down
circRNA_11673	0	−Inf	0.016	Down
circRNA_13282	0	−Inf	0.047	Down

**Figure 1:**
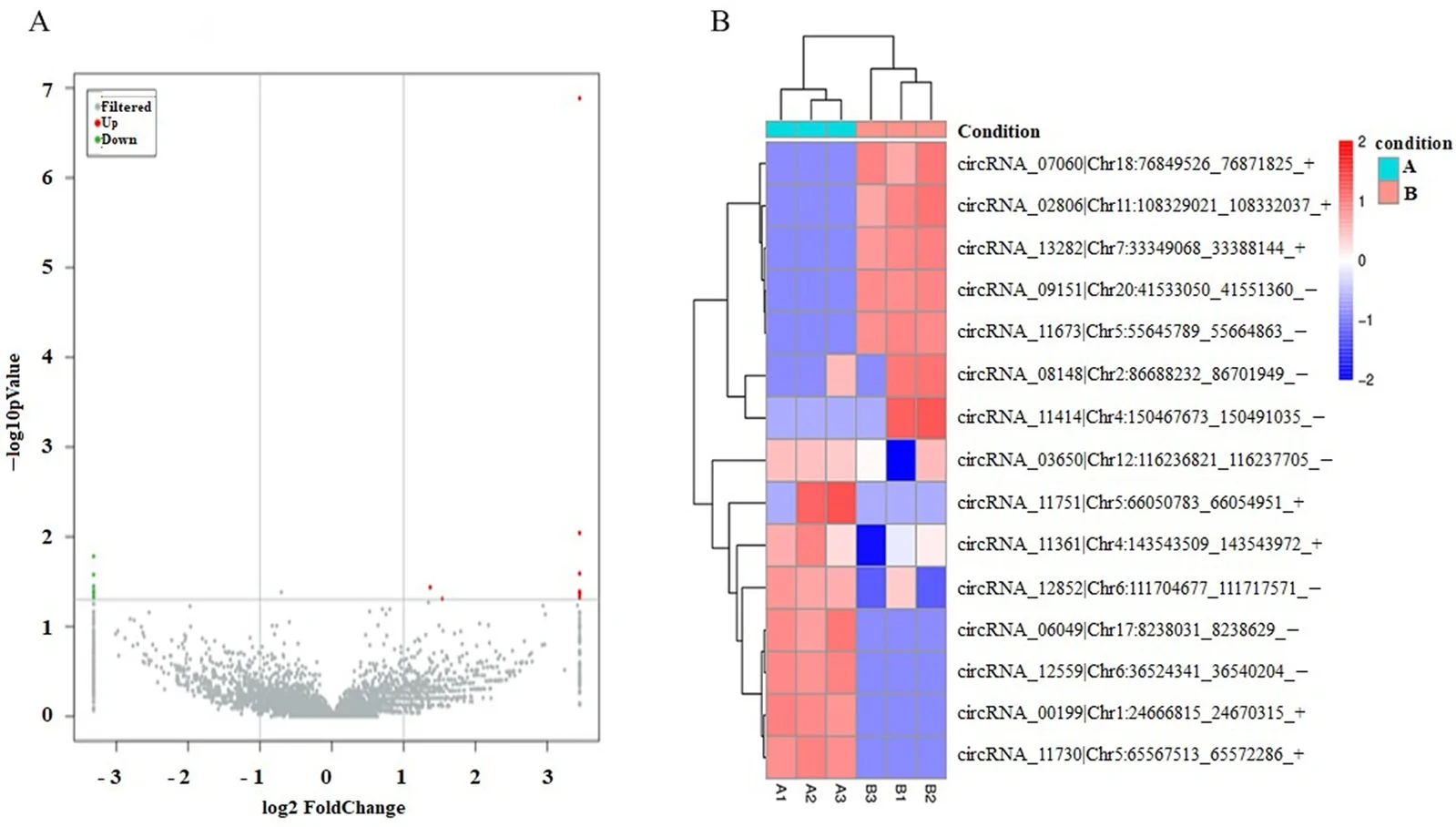
Differentially expressed circRNAs between AF-related stroke patients and AF patients. **(A)** Volcano plots to visualize the differential expression of circRNAs. The red and green points in the plot represent the statistically significant differentially expressed circRNAs. **(B)** Hierarchical cluster analysis of all deregulated circRNAs. circRNA, circular RNA.

### GO and KEGG pathway enrichment analyses of differentially expressed circRNAs

GO and KEGG pathway enrichment analyses of differentially expressed circRNAs were performed, and their roles were visually illustrated. A comparative plot of the distribution of the upregulated and downregulated differential circRNA maternal genes at the secondary level in GO analysis is shown in Figure 2A. The biological processes associated with the differentially expressed circRNAs were mainly enriched in cellular processes, biological regulation, metabolic processes, and responses to stimuli; at the cellular component level, these differentially expressed circRNAs were mainly localized to cells, cell parts, organelles, and organelle parts, while their molecular functions were mainly associated with binding and catalytic activity. The results of the KEGG pathway enrichment analysis are shown in Figure 2B. According to the KEGG results, the differentially expressed circRNA-related signaling pathways were primarily involved in signal transduction, infectious diseases, and immune system-related pathways.

**Figure 2:**
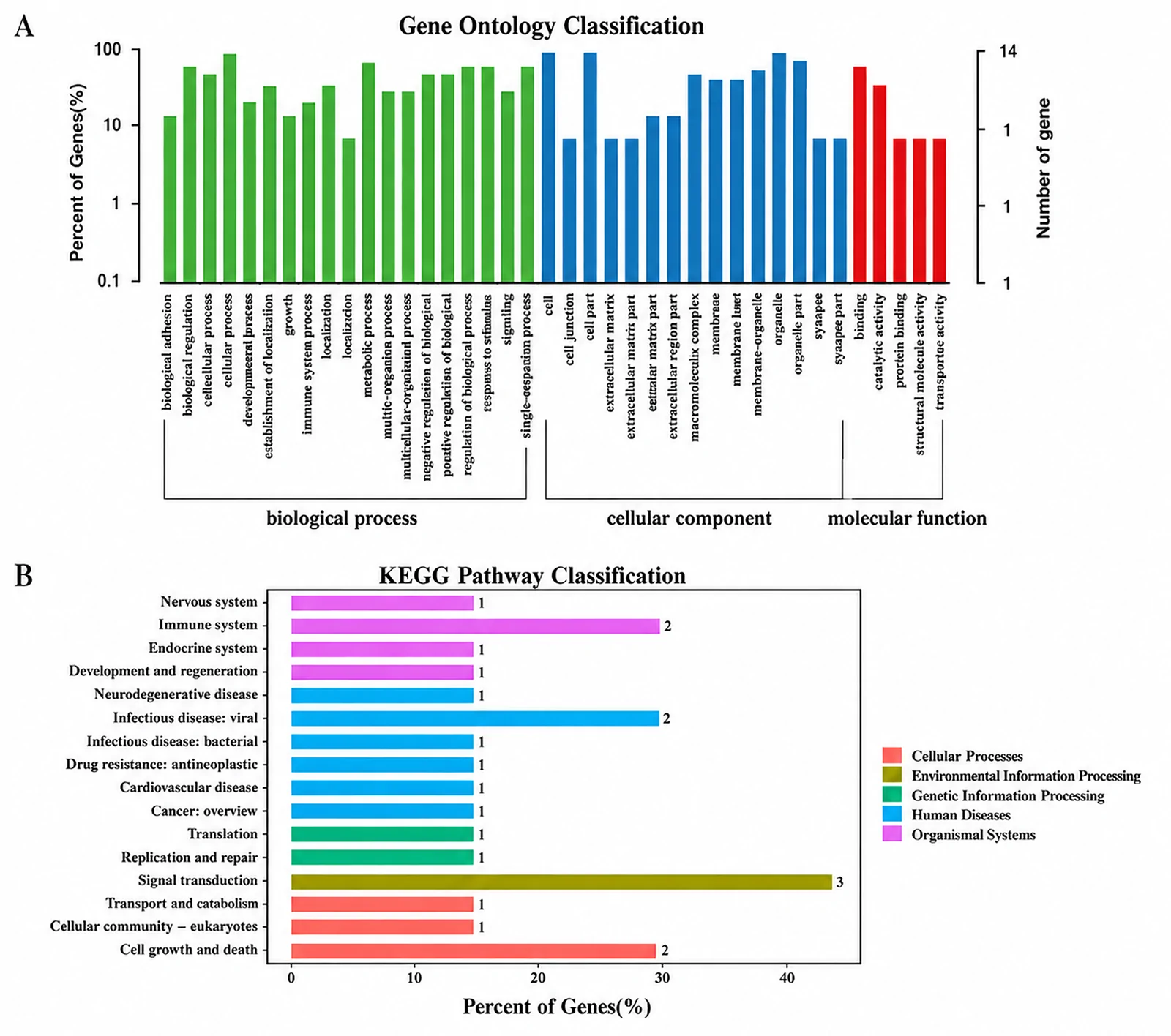
Results of GO and KEGG pathway analyses of differentially expressed circRNAs. **(A)** GO secondary level of differentially expressed circRNAs. Red indicates GO secondary entries enriched in upregulated differentially expressed circRNAs maternal genes, while green indicates GO secondary entries enriched in downregulated differentially expressed circRNAs maternal genes. **(B)** Results of KEGG pathway analysis of differentially expressed circRNAs.

### circRNA–miRNA co-expression network

Among the enrichment results for the differentially expressed circRNAs, the top 300 miRNA–circRNA interaction pairs with the smallest *p*-values were selected to construct a circRNA–miRNA target interaction network (Figure 3). As shown in Figure 3, circRNA_08148, circRNA_12852, and circRNA_00199 emerged as the top three circRNAs with the highest number of binding nodes in the co-expression network, suggesting that these three circRNAs may play important roles in the mechanism underlying AF-related stroke.

**Figure 3:**
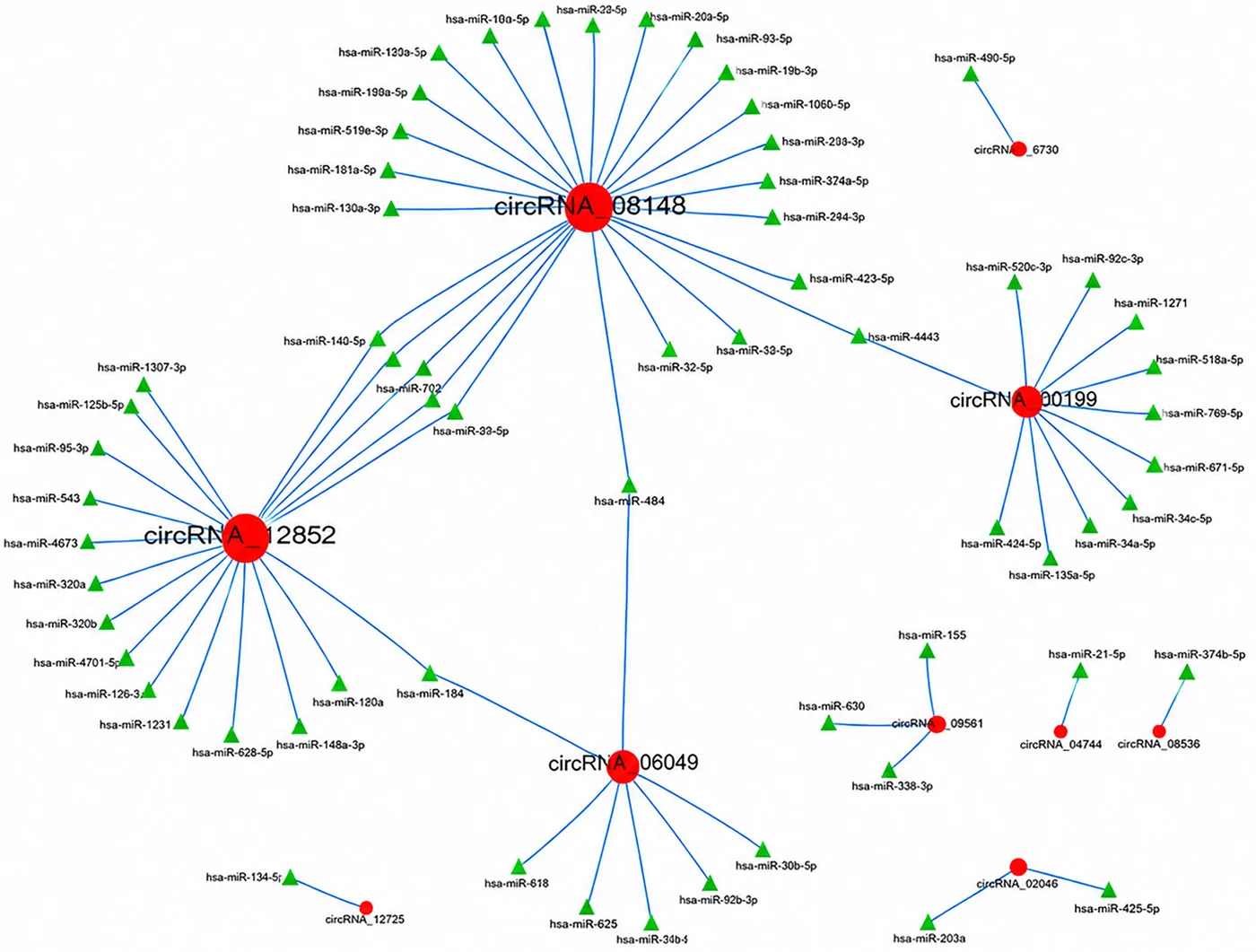
circRNA–miRNA co-expression network explored using Cytoscape. The red node represents a circRNA, and the green node represents an miRNA. circRNA_08148, circRNA_12852, and circRNA_00199 emerged as the top three circRNAs with the highest number of binding nodes in the co-expression network.

### qRT-PCR validation of differentially expressed circRNAs

Based on the microarray results, three upregulated circRNAs (circRNA_00199, circRNA_06049, and circRNA_12852) and three downregulated circRNAs (circRNA_08148, circRNA_11414, and circRNA_09151) were selected for qRT-PCR validation. The primer sequences for RT-PCR are listed in Table 3. As a result, three upregulated circRNAs (*p* < 0.05 or *p* < 0.01 for circRNA_00199, circRNA_06049, and circRNA_12852) and two of the three downregulated circRNAs (*p* < 0.05 or *p* < 0.01 for circRNA_11414 and circRNA_09151) showed significantly different expression levels between AF-related stroke patients and AF patients (Figure 4), consistent with the microarray results.

**Table 3:** Primer sequences for qRT-PCR validation.

Gene name	Forward primer sequences (5′–3′)	Reverse primer sequences (5′–3′)
circRNA_00199	AAAGGTGGCAAAATGGCTGC	TCTTTTTGGCGCTTTCGTGG
circRNA_06049	GAATTCCTCAGGGGAAGGGC	GGACACTCGCAGTTCTGTCA
circRNA_12852	TTCTCCTTCTCCTTTCAGTCAGG	AGGTCCCCGTATGAGACGAA
circRNA_08148	TCCTTCTGAATTGGCAGCGA	TTTACTGGGTGCTTTGCCCA
circRNA_11414	TGCCATGCAAGTGAGTCCAT	GCTGGTATGGCTGGTCTTGT
circRNA_09151	ACCAGCATGCTCAATCCCTT	TATGGCCGTGAGTGACATGG
GAPDH	GGAGCGAGATCCCTCCAAAAT	GGCTGTTGTCATACTTCTCATGG

**Figure 4:**
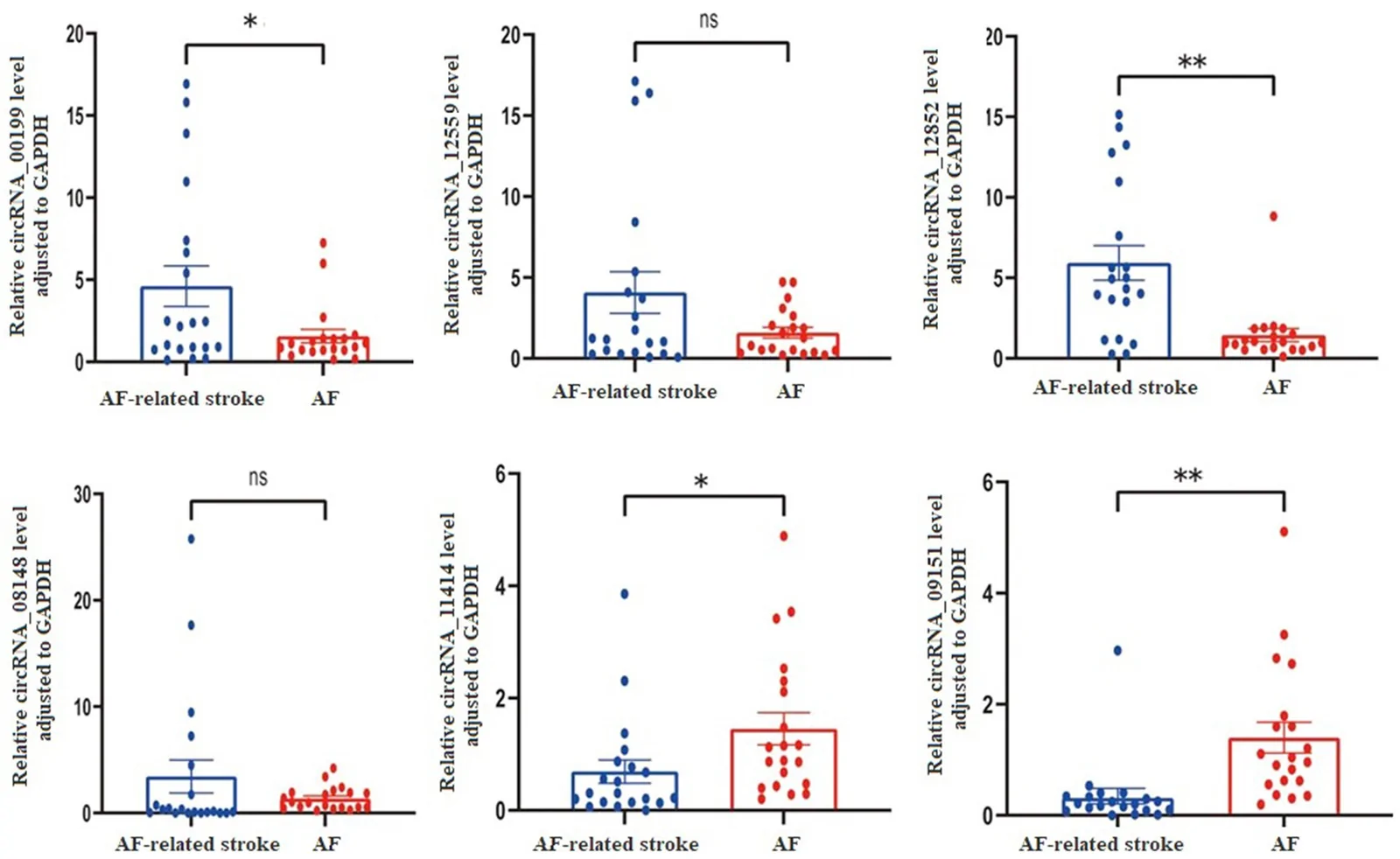
qRT-PCR validation of differentially expressed circRNAs. Compared with the control group, *p < 0.05, **p < 0.01.

### Binary logistic regression analysis

D-dimer and NLR showed statistically significant differences between the AF-related stroke and AF groups in the baseline comparison (*P* < 0.05), while circRNA_12852 was statistically significant in univariate binary logistic regression analysis (*P* < 0.05). These variables were subsequently included in the multivariate binary logistic regression analysis. As shown in Tables 4, 5, circRN_A12852 was found to be an independent risk factor for stroke in AF patients after adjusting for D-dimer and NLR (odds ratio (OR): 1.80, 95% CI: 1.150–2.817, *p*-value: 0.01).

**Table 4:** Results of univariate binary logistic regression.

Variable	OR	95% CI	*p*-value
D-dimer (µg/L)	1.00	1.000–1.001	0.275
NLR	1.32	0.953–1.828	0.095
circRNA_12852	1.70	1.133–2.550	0.010

NLR, neutrophil-to-lymphocyte ratio.

**Table 5:** Results of multivariate binary logistic regression.

Variable	OR	95% CI	*p*-value
D-dimer (µg/L)	1.00	1.000–1.000	0.276
NLR	1.33	0.920–1.931	0.128
circRNA_12852	1.80	1.150–2.817	0.010

NLR, neutrophil-to-lymphocyte ratio.

### ROC analysis of differentially expressed circRNAs

Using GraphPad Prism 9.0 software, ROC curves were plotted according to the relative quantitative values of circRNA_12852 (Figure 5). It was found that high expression of circRNA_12852 had some discriminative value for AF-related stroke (area under the receiver operating characteristic curve (AUC): 0.810, 95% CI: 0.662–0.958, *p*-value: 0.0008), and the maximum Youden index was calculated to be 0.7, with a cutoff value of 2.76, a sensitivity of 75%, a specificity of 95%, a positive predictive value of 93.8%, and a negative predictive value of 79.2%.

**Figure 5:**
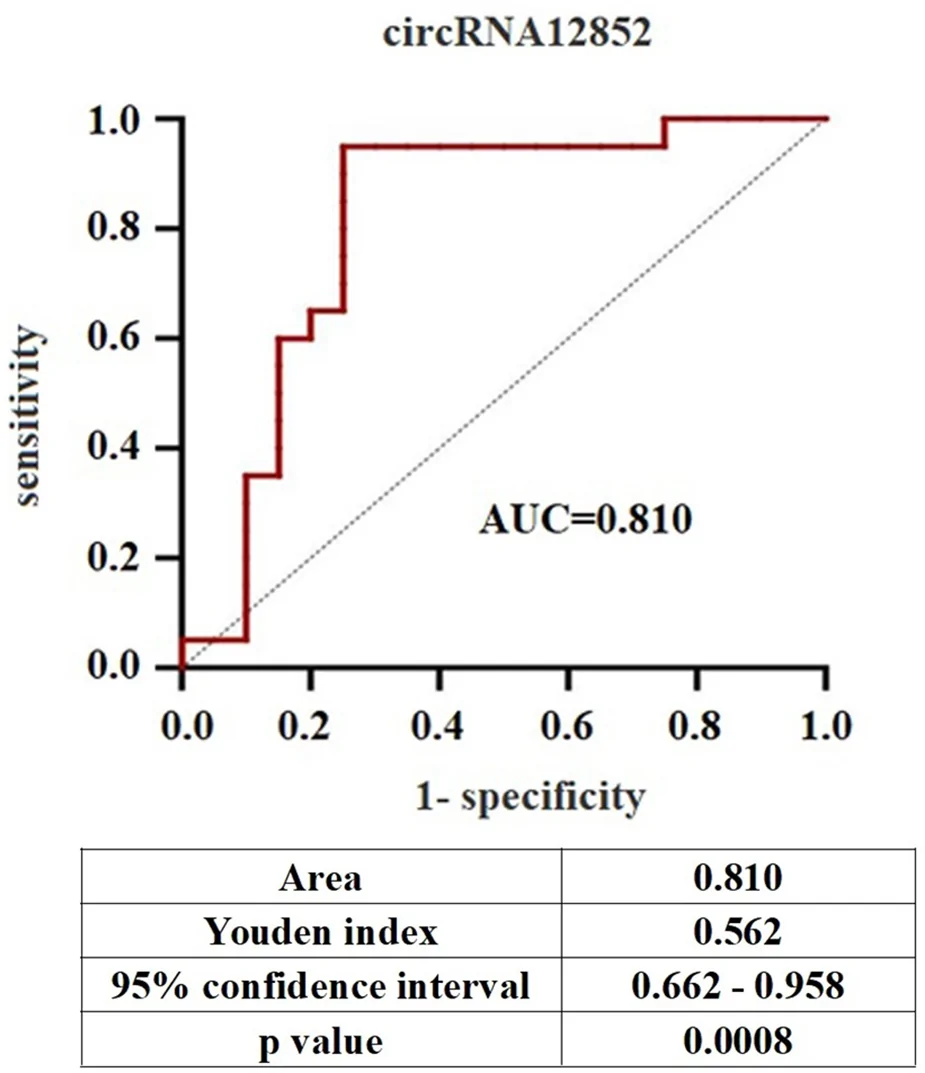
ROC analysis of circRNA_12852 for discriminating AF-related stroke.

## Discussion

The main findings of the present study were as follows: First, the expression of circRNAs in peripheral blood varied significantly between AF-related stroke patients and AF patients. Second, circRNA_08148, circRNA_12852, and circRNA_00199 emerged as the top three circRNAs with the highest number of binding nodes in the co-expression network. Third, circRNA_12852 was identified as an independent risk factor for AF-related stroke, providing a novel circulating biomarker to complement traditional risk stratification tools for AF-related stroke.

circRNAs have been shown to be associated with AF development and other AF-related diseases, such as coronary artery disease (12). In our study, compared with AF patients, 15 circRNAs were significantly differentially expressed in AF-related stroke patients. Furthermore, six differentially expressed circRNAs (circRNA_00199, circRNA_06049, circRNA_12852, circRNA_08148, circRNA_11414, and circRNA_09151) were selected for qRT-PCR validation in this study. Among these, three upregulated circRNAs (circRNA_00199, circRNA_06049, and circRNA_12852) and two downregulated circRNAs (circRNA_11414 and circRNA_09151) showed significantly different expression levels, consistent with the microarray results. These differentially expressed circRNAs may be used to predict the potential risk of stroke in patients with AF, which in turn reduces the risk of AF-related stroke through timely intervention and improves patient outcomes.

Inflammation, immune responses, and transduction play important roles in AF recurrence, maintenance, and stroke occurrence (13). Our KEGG enrichment analysis of the host genes of differentially expressed circRNAs revealed significant enrichment in pathways related to signal transduction, infectious disease, and the immune system, which aligns with prior transcriptomic findings describing circRNA-mediated activation of inflammatory signals in arrhythmia cohorts (13).

The sponge activity of circRNAs that bind specific miRNAs is an important molecular mechanism in the progression of AF (14). circRNAs exert biological functions primarily through miRNA sponge effects to modulate downstream signaling cascades, and accumulating evidence has confirmed their critical regulatory roles in AF-related inflammatory and thrombotic pathological processes. In the present study, construction of the circRNA–miRNA co-expression network showed that circRNA_08148, circRNA_12852, and circRNA_00199 emerged as the top three circRNAs with the highest number of binding nodes, suggesting that the three circRNAs might play important roles in the mechanism underlying AF-related stroke.

In the present study, binary logistic regression was performed to assess the discriminative efficacy of circRNAs for AF-related stroke. In the baseline comparison, D-dimer and NLR differed significantly between the AF-related stroke and AF groups, whereas circRNA_12852 was statistically significant in univariate binary logistic regression analysis. D-dimer, NLR, and circRNA_12852 were subsequently included in the multivariate binary logistic regression model. To isolate the independent predictive effect of circRNA_12852, we incorporated D-dimer and NLR into a multivariate binary logistic regression model to control for inflammatory and prothrombotic confounding factors. After adjusting for D-dimer and NLR, circRNA_12852 was found to be an independent risk factor for AF-related stroke.

Current mainstream risk scoring systems lack molecular markers reflecting real-time inflammatory and epigenetic risk of thrombosis (6). The results of the present study indicate that ciRNA_12852 could serve as a discriminative marker for AF-related stroke. This may be attributed to its stable, closed-loop structure, which is resistant to exonuclease degradation. Combined with conventional inflammatory and coagulation indicators (NLR, D-dimer), circRNA_12852 has the potential to refine risk stratification for patients with non-valvular AF, facilitate individualized anticoagulation decision-making, and enable early intervention for populations with hidden high embolic risk. Through statistical analysis and verification, the discriminative roles of circRNA_12852 in AF-related stroke are worth noting, although there may exist more circRNAs that could also have similar discriminative values.

## Limitations

The present study has several limitations. First, only 3 pairs of samples were used for whole-transcriptome sequencing, 6 genes were subjected to qRT-PCR validation, and only 20 pairs of samples were included in subsequent qRT-PCR verification. Larger patient cohorts are required to further validate the predictive value of circRNAs. Second, other established biomarkers linked to AF-related stroke, such as NT-proBNP and cardiac troponin I, were not incorporated into the analysis; these confounding factors may interfere with the experimental results. At present, relevant research specifically exploring circRNA function in AF-related stroke remains scarce, thereby restricting our in-depth interpretation of its upstream and downstream signaling axes. To address the above limitations and strengthen the reliability of our findings, a large-sample, multicenter prospective cohort study to externally verify the discriminative efficiency of circRNA_12852 should be performed. Furthermore, we will construct a comprehensive risk predictive nomogram that combines circRNA_12852, inflammatory markers, and thrombotic indicators and eliminates residual confounding bias to improve risk stratification accuracy for AF-related stroke patients. Third, cellular functional experiments and AF animal models will be conducted to explore how circRNA_12852 modulates immune and signal transduction pathways, identify its target miRNAs and downstream prothrombotic genes, and reveal the molecular mechanism by which it promotes cardiogenic stroke.

In general, our preliminary findings provide a novel epigenetic clue for identifying high-risk AF patients complicated by stroke. Subsequent large-scale clinical validation and mechanistic exploration will lay a solid foundation for the clinical application of circRNA_12852 in thromboembolic risk screening for AF.

## Data Availability

The original contributions presented in the study are included in the article/Supplementary Material. Further inquiries can be directed to the corresponding author.
